# Predictive role of HER2-status on the effectiveness of endocrine adjuvant treatment in postmenopausal breast cancer patients: a population-based cohort study

**DOI:** 10.1007/s10549-020-06022-5

**Published:** 2020-11-30

**Authors:** Aglaia Schiza, Davide Mauri, Irma Fredriksson, Anna-Karin Wennstig, Antonios Valachis

**Affiliations:** 1 Science for Life Laboratory, Department of Immunology, Genetics and Pathology, Uppsala University, Dag Hammarskjoldsvag 20, 751 85 Uppsala, Sweden; 2 Department of Oncology, Uppsala University Hospital, 751 85 Uppsala, Sweden; 3 Medical Oncology, University Hospital of Ioannina, Ioannina, Greece; 4 Department of Molecular Medicine and Surgery, Karolinska Institutet, Stockholm, Sweden; 5 Department of Breast and Endocrine Surgery, Karolinska University Hospital Solna, Stockholm, Sweden; 6 Department of Surgical and Perioperative Science, Umeå University, 901 85 Surgery, Umeå, Sweden; 7 Department of Oncology, Sundsvall Hospital, 85186 Sundsvall, Sweden; 8 Department of Oncology, Faculty of Medicine and Health, Örebro University, SE 70182 Örebro, Sweden

**Keywords:** Breast cancer, HER2-status, Hormone receptor-positive, Endocrine treatment, Adjuvant, Postmenopausal

## Abstract

**Purpose:**

There are conflicting results on the potential role of HER2-status on the efficacy of aromatase inhibitors (AIs) and tamoxifen (TAM) in patients with hormone receptor (HR)-positive breast cancer (BC). The purpose of this population-based cohort study was to investigate the potential benefit of AIs compared to TAM as adjuvant therapy in postmenopausal BC patients by HER2-status in the era of modern therapy with HER2-blockade.

**Methods:**

A population-based cohort study was performed including all postmenopausal women diagnosed with HR-positive BC without distant metastasis between 2007 and 2012 in three healthcare regions in Sweden. We analyzed the breast cancer-specific survival (BCSS) and overall survival (OS) in two distinct cohorts (HER2-negative, HER2-positive) based on the type of endocrine therapy (ET) used. A propensity score matching was performed separately in the HER2-negative and HER2-positive cohorts, respectively.

**Results:**

After propensity score matching, 4368 patients with HER2-negative and 214 patients with HER2-positive BC were available for analysis. In the HER2-negative cohort, an improved BCSS [Hazard Ratio (HR): 0.51; 95% confidence interval (CI): 0.34–0.77, *p* value < 0.001] and a trend toward improved OS (HR: 0.66; 95% CI: 0.41–1.08, *p* value = 0.093) in favor of AI-based therapy was observed. In the HER2-positive cohort, no statistically significant difference between AI-based ET and TAM was found in terms of either BCSS or OS, although the direction of HR was similar as in the HER2-negative cohort (HR for BCSS: 0.84; 95% CI: 0.14–5.04, *p* = 0.849; HR for OS: 0.62; 95% CI: 0.10–3.38, *p* = 0.345).

**Conclusion:**

Our study results, based on propensity-matched cohorts, did not support any predictive value of HER2-status on endocrine therapy in postmenopausal BC patients. AI-based ET remains the treatment of choice for postmenopausal BC patients with HR-positive disease in the modern era of HER2-directed therapy irrespective of HER2-status.

## Introduction

Breast cancer (BC) is a heterogeneous disease composed of various biologic subtypes with distinct behavior. Hormone receptor (HR)-positive [estrogen (ER) and/or progesterone (PgR) receptor-positive] BC comprises the most common type of BC, whereas amplification or overexpression of the human epidermal growth factor receptor 2 (HER2) oncogene is present in approximately 20% of invasive BC, half of which also express HR [1].

The cornerstone of adjuvant treatment in HR-positive BC is the endocrine therapy (ET) which has resulted in improved survival [2, 3]. For postmenopausal patients with HR-positive BC, both aromatase inhibitors (AIs) and tamoxifen (TAM) are valid treatment options but AIs have demonstrated superior efficacy compared to TAM [4]. In patients with HER2-positive invasive BC, HER2-directed therapy has altered the natural history of this aggressive subtype resulting in improved survival in all treatment settings [5, 6]. Patients with HR+/HER2 + BC are treated with both ET and HER2-directed therapy in adjuvant setting.

A complex molecular bi-directional crosstalk between HR and HER2 receptor pathways has been observed. In fact, in vitro studies have identified different mechanisms of resistance to both TAM and AIs mediated by the HR/HER2 crosstalk [7, 8]. In addition, clinical evidence supports the preclinical observations since an inverse relationship between HER2 overexpression and response to ET has been observed in different treatment settings [9–12].

Considering the different resistance mechanisms that are mediated by the HR/HER2 crosstalk for AIs and TAM, one could argue that there might be clinically significant differences on the efficacy of AIs and TAM, respectively, depending on the treatment sequence in HER2 + BC. This hypothesis is not supported by the results of the EBCTCG meta-analysis comparing the efficacy of AIs versus TAM in postmenopausal women where AIs proved to be more effective irrespective of HER2-status [3]. However, more than 70% of patients in this meta-analysis had an “unknown” HER2-status and most of the patients were not treated with HER2-directed therapy, thus jeopardizing the generalizability of the results. In a recent meta-analysis of three adjuvant randomized trials with postmenopausal early-stage BC, Bartlett et al. [13] showed that patients with HR+/HER2 *−* BC benefited more from upfront AI compared to TAM in the adjuvant setting, whereas upfront AI in HR+/HER2 + BC did not seem to provide any additional benefit compared to TAM. Though the meta-analysis of Bartlett overcame the limitation of “unknown” HER2-status through centrally confirmed HER2 analysis, most of the patients did not receive HER2-directed therapy. Dackus et al. provided data from a population-based cohort study indicating that postmenopausal patients with early-stage HR+/HER2 + BC may experience a small but, non-significant AI benefit. However, nearly 30% of the patients included in the analysis received no HER2-directed therapy [14].

Thus, conflicting clinical evidence about the potential impact of HER2-status on the efficacy of AIs compared to TAM do exist, whereas the inconsistencies in the treatment strategies regarding HER2-directed therapy among the current studies influence the generalizability of the results in the modern era. The aim of this population-based cohort study was to investigate whether the magnitude of potential benefit of AIs compared to TAM as adjuvant therapy in postmenopausal BC patients was different based on HER2-status in the modern era of HER2-directed therapy.

## Methods

### Study design and data sources

We performed a population-based cohort study using the BCBaSe database. BCBaSe is a research database consisting of the data linkage of The Regional Breast Cancer Quality Registries of the Uppsala/Örebro, Stockholm-Gotland and Northern regions of Sweden, the National Patient Register (information on hospital admission dates and diagnosis of diseases), the Swedish Cancer Register, the Swedish Cause of Death Register, the Swedish Prescribed Drug Register, all held by the National Board of Health and Welfare, and the Longitudinal Integration Database for Health Insurance and Labour Market Studies (LISA) and the Total Population Register, managed by Statistics Sweden. Information from these registers is linked using the ten-digit personal identifier numbers assigned for all citizens registered in Sweden. A detailed description of BCBaSe is presented elsewhere [14].

The datasets analyzed during the current study are available from the corresponding author on reasonable request.

### Identification of study cohort

Within the BCBaSe, we identified all postmenopausal women residing in the Uppsala/Örebro, Stockholm-Gotland, and Northern health care regions of Sweden who were diagnosed with HR-positive invasive BC (regardless of HER2-status) without distant metastasis between January 1, 2007, and December 31, 2012. We excluded patients with unknown HER2-status, patients without ET registered, as well as patients with HER2-positive BC who did not receive trastuzumab as a part of neo-/adjuvant therapy. The type of ET was collected based on the planned treatment according to physicians' prescriptions. Nearly all patients were planned to receive 5 years endocrine treatment since extended endocrine therapy was not recommended by the Swedish National Guidelines during the study period.

During the study period ovarian function suppression (OFS) was an uncommon treatment choice for breast cancer patients as it was not recommended by the Swedish national guidelines. In any case, premenopausal patients who had addition of OFS to ET between 2007 and 2012 were not included in our study cohort. Since HER2-targeted adjuvant treatment became standard after 2005, only data after 2007 and later on were used to in the present study to reduce eventual treatment bias since HER2-targeted treatment were not uniformly available before that time.

### Definitions

HR-positive BC was defined as estrogen receptor ≥10% and/or progesterone receptor ≥10% according to the Swedish National Treatment Guidelines on BC. HER2 was defined as positive if HER2 was categorized as 3+ by immunohistochemistry analysis (IHC) or in IHC 2+ with amplification of the HER2 gene verified through situ hybridization.

The Charlson Comorbidity Index (CCI) was used to categorize patients according to the presence of co-morbidities.

The patients treated with ET were categorized as: TAM-treated when patients received only TAM; AI-based treated when the patients received AI only or sequential treatment with AI and TAM in any sequence.

### Outcomes of interest

The outcomes of interest were breast cancer-specific survival (BCSS) defined as the time from BC diagnosis to death due to BC, and overall survival (OS) defined as the time from BC diagnosis to death due to any cause, in the two distinct cohorts (HER2-negative and HER2-positive cohorts).

### Statistical analysis

The continuous variables were expressed by median and interquartile rate (IQR), whereas the categorical variables were expressed by number and percentage of patients in each category.

The Chi-square test was used for the bivariate analyses between type of endocrine treatment (TAM vs. AI-based) and patient- or tumor characteristics within two separate cohorts based on HER2-status.

To minimize the potential effect of confounding factors on the choice of ET, we used a propensity score matching (PSM) approach separately for the HER2-positive and HER2-negative cohorts to create more balanced ET groups. The propensity score (PS) was estimated using multivariate logistic regression model with the type of ET as dependent variables and the variables with statistically significant difference (*P* value < 0.05) between the ET groups in the bivariate analyses as covariates. The variables with >10% missing values were excluded from the models to maintain a good level of matching since the analyses were performed using the complete case approach. The following variables were included as covariates: age, type of surgery, histology, anatomic stage, PgR-status, CCI, and chemotherapy use.

A 1:1 PSM was performed using the nearest-neighbor matching method without replacement and a caliper width of 0.1. Post-match comparison between ET group and patient- or tumor characteristics was performed using the standardized differences (SD). A SD below 0.1 was reliable enough to provide well-balanced characteristics after matching.

The analysis of time-to-event (BCSS, OS) outcomes among the PS-matched cases was performed by the Kaplan–Meier method and tested by log-rank test. Cox proportional hazards regression models were used to investigate whether there is an independent association between type of ET and BCSS or OS adjusting for variables associated with BCSS or OS, respectively (with a *P* value < 0.05) according to univariate Cox analyses.

All statistical analyses were performed using the Statistical Package for the Social Sciences (SPSS) (version 24.0; SPSS, Inc., Chicago, IL, USA), and *p* < 0.05 were considered statistically significant.

## Results

### Study cohort

**Fig. 1:**
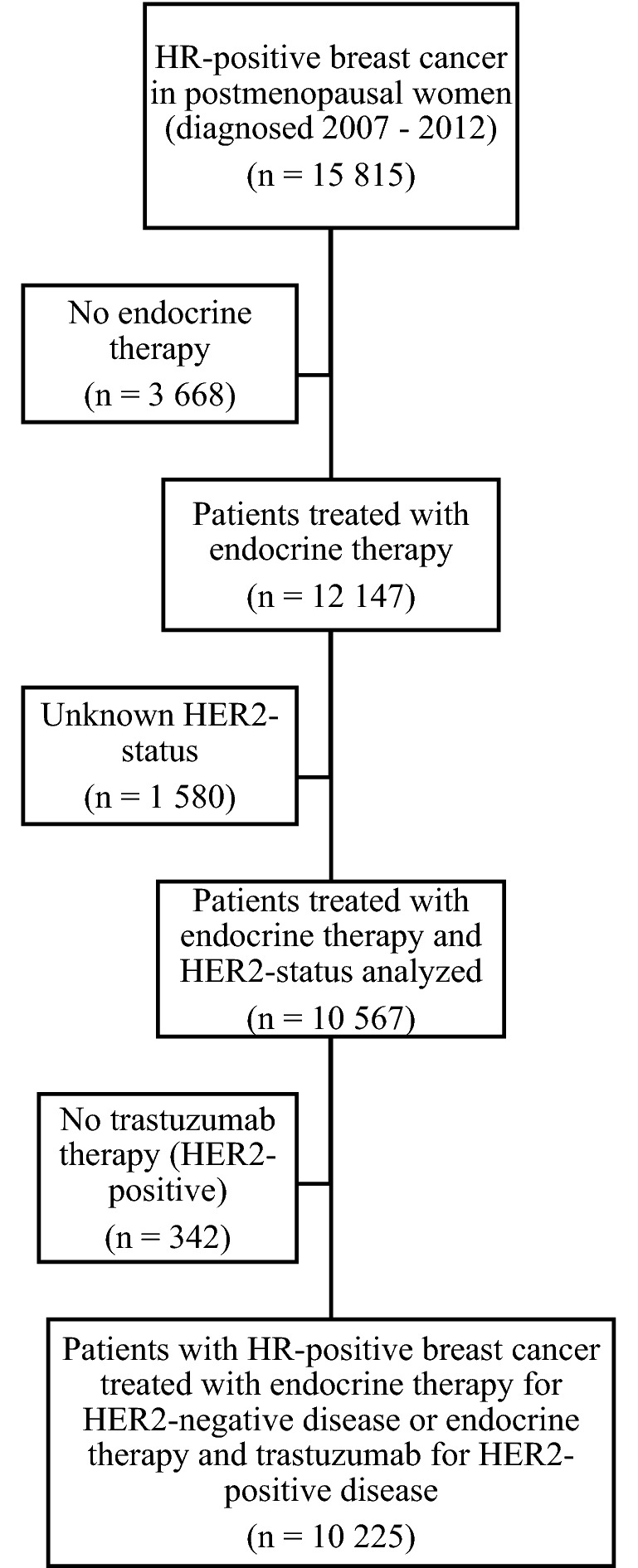
Flowchart diagram of study cohort selection

From a total of 15,815 patients in BCBaSe, 10,225 were eligible for the study cohort after applying the inclusion and exclusion criteria (Fig. 1). These patients were divided into a HER2-negative cohort (*n* = 9543) and a HER2-positive cohort (*n* = 682). After propensity score matching, 4368 patients in the HER2-negative cohort and 214 patients in the HER2-positive cohort remained available for analyses.

The median follow-up was 44 months in the HER2*-*negative cohort (IQR: 33) and 42 months in the HER2-positive cohort (IQR: 32).

### Baseline characteristics

Data on patient-, tumor and treatment characteristics in the HER2-negative cohort before and after matching are shown in Table 1. Before matching, AI-based ET was associated with more advanced age and negative prognostic factors as advanced stage, PgR-negative disease, grade III disease, and with more extensive adjuvant treatment (chemotherapy and radiotherapy). After propensity matching, age and use of radiotherapy remained statistically significantly associated with AI-based ET, but not the other above-mentioned variables.

**Table 1:** Patient-, tumor and treatment characteristics in unmatched and propensity-matched patients with HER2-negative breast cancer

Characteristics	Before matching			After matching		
	Tamoxifen (*n* = 4680)	AI-based (*n* = 4863)	*P* value	Tamoxifen (*n* = 2184)	AI-based (*n* = 2184)	*P* value
Age ≤ 60 61–65 66–70 71–75 > 75	1218 (26.0) 1092 (23.3) 950 (20.3) 586 (12.5) 834 (17.8)	1131 (23.3) 1069 (22.0) 954 (19.6) 673 (13.8) 1036 (21.3)	<0.001	608 (27.8) 479 (21.9) 328 (15.0) 266 (12.2) 503 (23.0)	503 (23.0) 503 (23.0) 458 (21.0) 297 (13.6) 423 (19.4)	<0.001
Detection method Screening Clinically detected Missing	2638 (56.4) 1177 (25.1) 865 (18.5)	2207 (45.4) 1375 (28.3) 1281 (26.3)	<0.001	NA	NA	NA
Type of surgery Breast conserving Mastectomy	3335 (71.3) 1345 (28.7)	2446 (50.3) 2417 (49.7)	<0.001	1307 (59.8) 877 (40.2)	1376 (63.0) 808 (37.0)	0.032
Histology Ductal or mixed Lobular Other Missing	3875 (82.8) 489 (10.4) 298 (6.4) 18 (0.4)	4243 (87.3) 516 (10.6) 87 (1.8) 17 (0.3)	<0.001	1780 (81.5) 90 (4.1) 314 (14.4) 0 (0)	1894 (86.7) 59 (2.7) 231 (10.6) 0 (0)	<0.001
Stage I II III Missing	3262 (69.7) 1099 (23.5) 127 (2.7) 192 (4.1)	1264 (26.0) 2563 (52.7) 875 (18.0) 161 (3.3)	<0.001	1070 (49.0) 997 (45.7) 117 (5.4) 0 (0)	1007 (50.7) 934 (42.8) 143 (6.5) 0 (0)	0.071
PgR-status Negative Positive Missing	681 (14.6) 3970 (84.8) 29 (0.6)	1011 (20.8) 3808 (78.3) 44 (0.9)	<0.001	416 (19.0) 1768 (81.0) 0 (0)	403 (18.5) 1781 (81.5) 0 (0)	0.614
Grade I II III Missing	1395 (29.8) 2414 (51.6) 332 (7.1) 539 (11.5)	658 (13.5) 2309 (47.5) 1158 (23.8) 738 (15.2)	<0.001	NA	NA	NA
CCI 0 1 2 3+	3959 (84.6) 373 (8.0) 244 (5.2) 104 (2.2)	3916 (80.5) 483 (9.9) 295 (6.1) 169 (3.5)	<0.001	1707 (78.2) 247 (11.3) 151 (6.9) 79 (3.6)	1799 (82.4) 201 (9.2) 119 (5.4) 65 (3.0)	0.006
Chemotherapy No Yes	4220 (90.2) 460 (9.8)	2924 (60.1) 1939 (39.9)	<0.001	1751 (80.2) 433 (19.8)	1711 (78.3) 473 (21.7)	0.136
Radiotherapy No Yes Missing	1227 (26.2) 3276 (70.0) 177 (3.8)	1006 (22.7) 3531 (72.6) 226 (4.6)	<0.001	736 (33.7) 1448 (66.3) 0 (0)	567 (26.0) 1617 (74.0) 0 (0)	<0.001
Educational level Low Middle High Missing	1310 (28.0) 1842 (39.4) 1485 (31.7) 43 (0.9)	1440 (29.6) 1913 (39.3) 1454 (29.9) 56 (1.2)	0.111	632 (28.9) 866 (39.7) 686 (31.4) 0 (0)	650 (29.8) 858 (39.3) 676 (31.0) 0 (0)	0.834
Civil-status Single Married Divorced Widow Missing	577 (12.3) 2369 (50.3) 939 (20.1) 795 (19.0) 0 (0)	579 (11.7) 2471 (50.8) 956 (19.7) 851 (17.5) 6 (0.1)	0.152	273 (12.5) 1068 (48.9) 438 (20.1) 405 (18.5) 0 (0)	267 (12.2) 1113 (51.0) 462 (21.2) 342 (15.7) 0 (0)	0.174
Household income Lowest group Medium lowest Medium highest Highest group Missing	1133 (24.2) 1199 (25.6) 1149 (24.6) 1185 (25.3) 14 (0.3)	1222 (25.1) 1185 (24.4) 1235 (25.4) 1196 (24.6) 25 (0.5)	0.322	574 (26.3) 573 (26.2) 490 (22.4) 547 (25.0) 0 (0)	527 (24.1) 526 (24.1) 585 (26.8) 546 (25.0) 0 (0)	0.159

Characteristics in HER2-positive cohort before and after matching are shown in Table 2. Before matching, AI-based ET was associated with more advanced age and stage. After propensity score matching no statistically significant differences in patient or treatment characteristics between the two groups were observed.

**Table 2:** Patient-, tumor and treatment characteristics in unmatched and propensity-matched patients with HER2-positive breast cancer

Characteristics	Before matching			After matching		
	Tamoxifen (*n* = 124)	AI-based (*n* = 558)	*P* value	Tamoxifen (*n* = 107)	AI-based (*n* = 107)	*P* value
Age ≤ 60 61–65 66–70 71–75 >75	67 (54.0) 26 (21.0) 13 (10.5) 13 (10.5) 5 (4.0)	191 (34.2) 138 (24.7) 131 (23.5) 72 (12.9) 26 (4.7)	0.001	57 (53.3) 25 (23.4) 12 (11.2) 9 (8.4) 4 (3.7)	58 (54.2) 23 (21.5) 12 (11.2) 11 (1.03) 3 (2.8)	0.980
Detection method Screening Clinically detected Missing	72 (58.1) 31 (25.0) 21 (16.9)	278 (49.8) 144 (25.8) 136 (24.4)	0.437	NA	NA	
Type of surgery Breast conserving Mastectomy	79 (63.7) 45 (36.3)	271 (48.6) 287 (51.4)	0.002	34 (31.8) 73 (68.2)	32 (29.9) 75 (70.1)	0.767
Histology Ductal or mixed Lobular Other Missing	113 (91.1) 5 (4.0) 2 (1.6) 4 (3.2)	520 (93.2) 22 (3.9) 3 (0.5) 13 (2.3)	0.438	101 (94.4) 1 (0.9) 5 (4.7) 0 (0)	104 (97.2) 1 (0.9) 2 (1.9) 0 (0)	0.851
Stage I II III Missing	58 (46.8) 58 (46.8) 7 (5.6) 1 (0.8)	195 (34.9) 251 (45.0) 97 (17.4) 15 (2.7)	0.002	51 (47.7) 41 (45.8) 7 (6.5) 0 (0)	54 (50.5) 45 (42.1) 8 (7.5) 0 (0)	0.851
PgR-status Negative Positive Missing	52 (41.9) 67 (54.0) 5 (4.0)	319 (57.2) 226 (40.5) 13 (2.3)	0.655	48 (44.9) 59 (55.1) 0 (0)	46 (43.0) 61 (57.0) 0 (0)	0.783
Grade I II III Missing	4 (3.2) 38 (30.6) 57 (46.0) 25 (20.2)	15 (2.7) 192 (34.3) 261 (46.8) 90 (16.1)	0.836	NA	NA	
CCI 0 1 2 3+	110 (88.7) 8 (6.5) 5 (4.0) 1 (0.8)	487 (87.3) 40 (7.2) 24 (4.3) 7 (1.3)	0.962	93 (86.9) 8 (7.5) 5 (4.7) 1 (0.9)	96 (89.7) 4 (3.7) 4 (3.7) 3 (2.8)	0.477
Chemotherapy No Yes	0 (0) 124 (100)	0 (0) 558 (100)	NC	0 (0) 107 (100)	0 (0) 107 (100)	NC
Radiotherapy No Yes Missing	16 (12.9) 102 (82.3) 6 (4.8)	97 (17.4) 447 (80.1) 14 (2.5)	0.264	16 (15.0) 91 (85.0) 0 (0)	16 (15.0) 91 (85.0) 0 (0)	1.000
Educational level Low Middle High Missing	25 (20.2) 61 (49.2) 38 (30.6) 0 (0)	129 (23.1) 254 (45.5) 172 (30.8) 3 (0.5)	0.710	25 (23.4) 49 (45.8) 33 (30.8) 0 (0)	24 (22.4) 48 (44.9) 35 (32.7) 0 (0)	0.956
Civil-status Single Married Divorced Widow	30 (24.2) 63 (50.8) 17 (13.7) 14 (11.3)	75 (13.4) 309 (55.4) 118 (21.1) 56 (10.0)	0.112	23 (21.5) 53 (49.5) 17 (15.9) 14 (13.1)	16 (15.0) 59 (55.1) 22 (20.6) 10 (9.3)	0.410
Household income Lowest group Medium lowest Medium highest Highest group Missing	15 (12.1) 33 (26.6) 35 (28.2) 40 (32.3) 1 (0.8)	92 (16.5) 130 (23.3) 162 (29.0) 171 (30.6) 3 (0.5)	0.610	15 (14.0) 25 (23.4) 31 (29.0) 36 (33.6) 0 (0)	16 (15.0) 26 (24.3) 26 (24.3) 39 (36.4) 0 (0)	0.894

### Breast cancer-specific survival

**Fig. 2:**
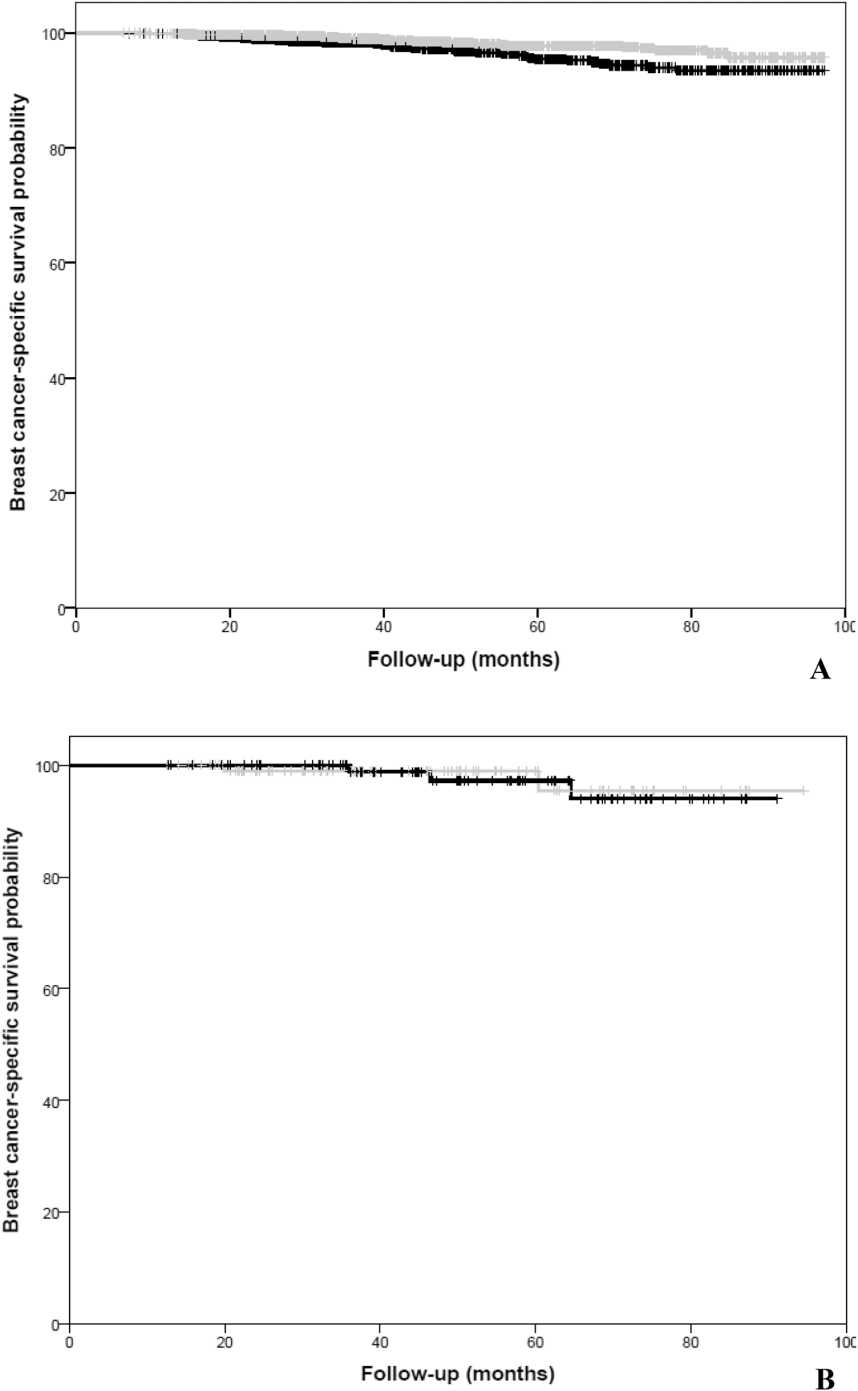
**a** Kaplan–Meier for breast cancer-specific survival in the HER2-negative cohort by type of endocrine therapy. **b** Kaplan–Meier for breast cancer-specific survival in the HER2-positive cohort by type of endocrine therapy. Gray line is AI-based therapy, whereas black line is Tamoxifen

In the HER2-negative cohort, AI-based ET was associated with improved BCSS compared to TAM in the univariate Cox regression analysis (HR: 0.51; 95% CI: 0.34–0.77, *p* < 0.001; Fig. 2a).

Multivariate Cox regression for BCSS (adjusted for age, stage, histology, PgR-status, chemotherapy, radiotherapy, type of surgery, CCI, civil-status) showed a similar improvement in BCSS in favor of AI-based ET as in the univariate analysis (HR: 0.54; 95% CI: 0.31–0.93, *p* = 0.032).

In the HER2-positive cohort, AI-based ET was not associated with improved BCSS compared to TAM in the univariate Cox regression analysis (HR: 0.84; 95% CI: 0.14–5.04), *p* = 0.849; Fig. 2b). Neither did the multivariate Cox regression for BCSS show any statistically significant improved BCSS [HR: 0.90 (95% CI: 0.15–5.45, *p* = 0.422)].

### Overall survival

**Fig. 3:**
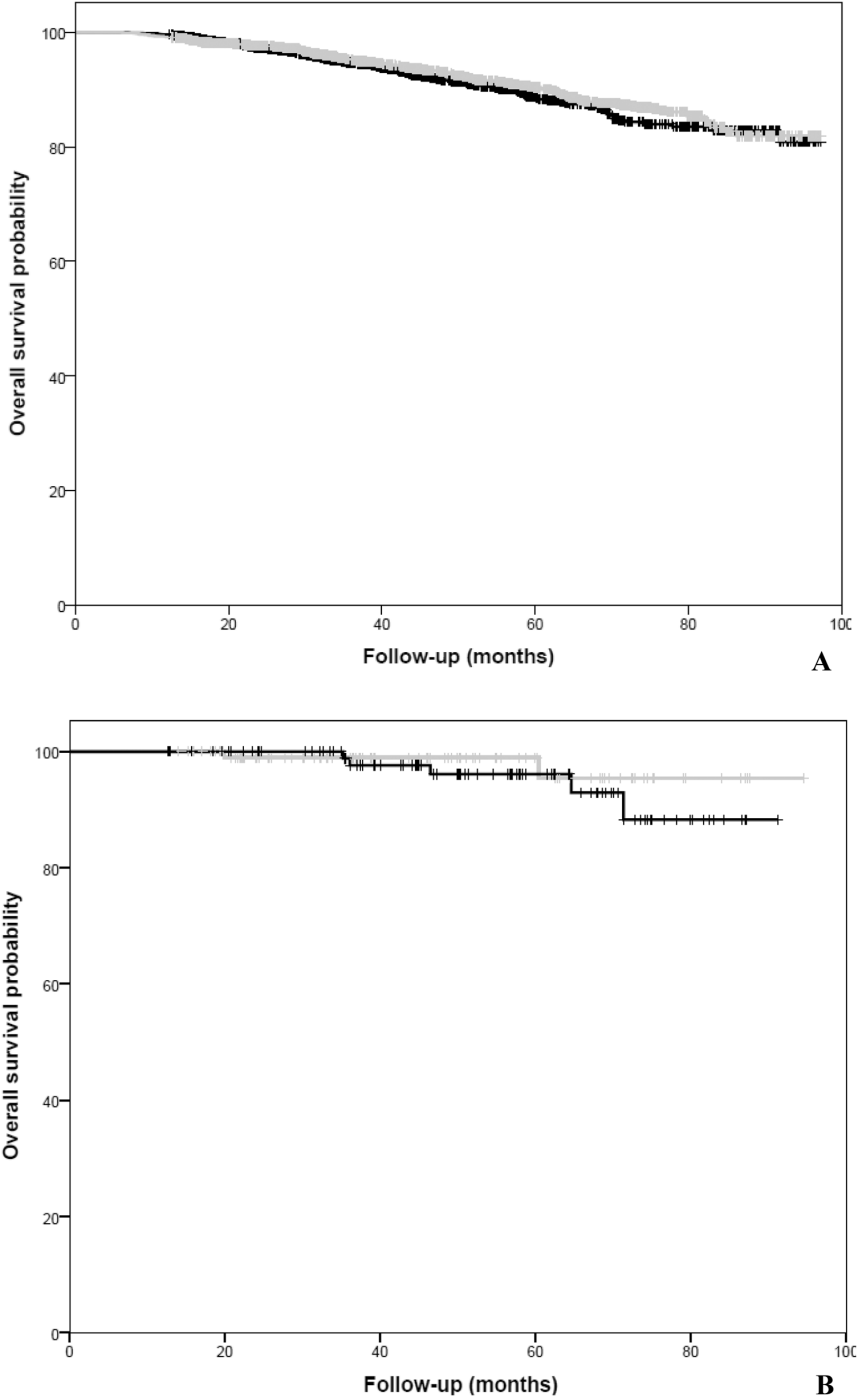
**a** Kaplan–Meier for overall survival in the HER2-negative cohort by type of endocrine therapy. **b** Kaplan–Meier for overall survival in the HER2-positive cohort by type of endocrine therapy. Gray line is AI-based therapy, whereas black line is Tamoxifen.

In the HER2-negative cohort, no statistically significant difference in OS between AI-based ET and TAM was observed in the univariate Cox regression analysis (HR: 0.89; 95% CI 0.73–1.10, *p* = 0.290; Fig. 3a). In multivariate Cox regression analysis (adjusted for age, stage, histology, PgR-status, chemotherapy, radiotherapy, type of surgery, CCI, civil-status education level, household income) we find a numerically but statistically non-significant improvement in OS in favor of AI-based ET (HR: 0.66; 95% CI: 0.41–1.08, *p* = 0.093).

In the HER2-positive cohort, AI-based ET was not associated with improved OS compared to TAM, neither in the univariate (HR: 0.50; 95% CI: 0.10–2.60, *p* = 0.413; Fig. 3b) nor in the multivariate Cox regression analysis (HR: 0.62; 95% CI: 0.11–3.38, *p* = 0.345).

## Discussion

In our study cohort of BC patients treated according to modern strategies, we found that AI-based therapy improves BCSS in the HER2-negative BC cohort. Although HR in the HER2-positive BC cohort is in the same direction as in HER2-negative BC cohort, the magnitude of effect seems to be larger for the HER2-negative cohort. Whether the non-statistically significant results for HER2-positive BC cohort is due to few events or a small and probably clinical non-significant impact of AI-based ET in HER2-positive disease remains to be answered.

Optimizing treatment strategies for HR+/HER2 + BC in postmenopausal women is an ongoing challenge in the modern era of HER2-directed therapy. Adjuvant trastuzumab, in conjunction with chemotherapy, is the standard of care for patients with HER2-positive disease [5]. The use of ET in this patient group is an important treatment approach but the increased risk for resistance to ET due to the crosstalk between HR and HER2 pathways poses challenges on which treatment approach that target both of these pathways is better in improving survival in this patient group [9, 10, 12].

In the present study, we investigated whether the benefit of AIs compared to TAM as adjuvant therapy in postmenopausal BC patients differed by HER2-status. Our study cohort reflects the current clinical practice in a real-world setting in Sweden where patients with HER2-positive BC received treatment in accordance to current clinical practice, i.e., modern HER2-directed therapy combined with ET.

In the HER2-negative cohort, we found a BCSS benefit in patients treated with AI-based therapy compared to TAM. Moreover, a trend for OS improvement was observed. This finding was expected considering evidence from randomized trials and meta-analyses about the efficacy of AI compared to TAM in HR+/HER2 − BC patients [2–4].

In the HER2-positive cohort, we observed a similar trend regarding the benefit of AI-based ET compared to TAM but the findings were not statistically significant. These findings are consistent with the results published by Darkus et al. [14] where postmenopausal patients with early-stage HR+/HER2 + BC seemed to experience a small; however, non-significant benefit from AI. Bartlett et al. [13] however found an opposite trend in favor of TAM in patients with HR+/HER2+ disease in their meta-analysis. The results of the EBCTCG meta-analysis showed that the rate ratios for improvement of OS in favor of AIs range between 0.84 and 0.89 in postmenopausal women [13]. Our results indicate a larger magnitude of benefit for AIs compared to TAM for OS. Although the magnitude of benefit seemed to be larger in our study results than in EBCTCG meta-analysis, this could be explained by the total number of events and the sample size in our cohort compared to the meta-analysis. In fact, our sample size was considerably smaller with one third of patients in the HER2-negative cohort and a fifth of patients in the HER2-positive cohort compared to the EBCTCG one. Moreover, a low number of events was captured in our study cohort due to the relatively short median follow-up. The substantial differences on the treatment approaches regarding HER2-directed therapy between the current study and the previous ones can partially explain the different results. All patients with HER2-positive disease in our study cohort received treatment with HER2-blockade, whereas in the ECBTCG- meta-analysis and in Bartlett et al. meta-analysis the majority of the patients received no HER2-blockade and in the Darcus et al. study cohort only two-thirds of patients received HER2-blockade.

In the multivariate analyses we found a larger magnitude of benefit for OS compared to the benefit on BCSS for the HER2-negative cohort. Although one could argue that this difference in magnitude seems to be paradoxical since the non-BC mortality is not expected to be reduced by ET and the risk for potentially ET-related toxicity is always present, this larger magnitude for OS should be considered a consequence of the few events in our cohort rather than a true difference in BCSS and OS benefits.

Our study has several limitations that needs to be considered when interpreting the results. First, type of ET was offered on selected grounds. AI-based therapy tended to be the treatment of choice in more advanced disease. Although this bias could be mitigated through PSM, the comparison between AI and TAM might still be influenced by differences in baseline characteristics. Moreover, we divided the patients into the treatment groups based on the planned treatment and not the actual treatment based on adherence. However, the adherence in Sweden is relatively good without any significant differences in adherence rates between AI and Tam and, thus the potential impact of this limitation to the results is expected to be low [15]. In addition, the sample size in the HR+/HER2 + BC cohort was relatively small, especially after PSM, thus jeopardizing the precision of the estimates. Furthermore, the median follow-up for the study cohort was relatively short resulting in a relatively low number of events. As a result, our findings can be considered valid regarding the first years after BC diagnosis but should not be applied in terms of late recurrence events.

In summary, our results do not support the hypothesis that HER2-status has a predictive value for ET in postmenopausal BC patients. AI-based ET remains the treatment of choice for postmenopausal BC patients in both HER2-negative and HER2-positive disease. Large-scale studies with longer follow-up would further enhance the evidence on the optimal ET strategy in HR+/HER2 + BC patients.

## Abbreviations

AIs: Aromatase inhibitors
BC: Breast cancer
BCSS: Breast cancer-specific survival
CCI: Charlson Comorbidity Index
ER: Estrogen receptor
ET: Endocrine therapy
*HER2*: Human epidermal growth factor receptor 2
HR: Hormone receptor
IHC: Immunohistochemistry analysis
IQR: Interquartile rate
OFS: Ovarian function suppression
PgR: Progesterone receptor
PS: Propensity score
PSM: Propensity score matching
SD: Standardized differences
TAM: Tamoxifen
OS: Overall survival
